# Population Genetic Analysis of *Lobelia rhynchopetalum* Hemsl. (Campanulaceae) Using DNA Sequences from *ITS* and Eight Chloroplast DNA Regions

**DOI:** 10.1100/2012/276451

**Published:** 2012-01-04

**Authors:** Mulatu Geleta, Tomas Bryngelsson

**Affiliations:** Department of Plant Breeding and Biotechnology, Swedish University of Agricultural Sciences, P.O. Box 101, 230 53 Alnarp, Sweden

## Abstract

DNA sequence data from the internal transcribed spacer of nuclear ribosomal DNA and eight chloroplast DNA regions were used to investigate haplotypic variation and population genetic structure of the Afroalpine giant lobelia, *Lobelia rhynchopetalum.* The study was based on eight populations sampled from two mountain systems in Ethiopia. A total of 20 variable sites were obtained, which resulted in 13 unique haplotypes and an overall nucleotide diversity (ND) of 0.281 ± 0.15 and gene diversity (GD) of 0.85 ± 0.04. Analysis of molecular variance (AMOVA) revealed a highly significant variation (*P* < 0.001) among populations (*F*_ST_), and phylogenetic analysis revealed that populations from the two mountain systems formed their own distinct clade with >90% bootstrap support. Each population should be regarded as a significant unit for conservation of this species. The primers designed for this study can be applied to any *Lobelia* and other closely related species for population genetics and phylogenetic studies.

## 1. Introduction

*Lobelia* is the largest genus within the subfamily Lobelioideae of Campanulaceae family comprising over 350 species that range from small herbs to giant woody plants.* Lobelia rhynchopetalum* Hemsl., which belongs to the subgenus Tupa, section Rhynchopetalum [1], is one of the 21 giant lobelia species of eastern Africa that represent a premier botanical example of spectacular evolutionary radiations [2, 3]. It belongs to the most famous giant lobelia group exhibiting a giant-rosette growthform [3]. Giant lobelias predated the formation of tall mountains in eastern Africa, and most evolution occurred in parallel up the mountains [4]. These authors also suggested that an extinct forest species gave rise to several alpine giant lobelias, possibly from which *L. rhynchopetalum* has evolved. *L. rhynchopetalum* and other giant lobelias of eastern Africa have the same chromosome number (2*n* = 28) as that of their progenitor [5, 6] and, thus, their evolution has occurred without a change in chromosome number. 

*L. rhynchopetalum* is a monocarpic perennial species endemic to the Ethiopian drained sites of the Afroalpine ecoregion (e.g. [1, 3]). The plant is frost tolerant and has an up to two-meter-tall unbranched stem with a large pith and thick and leathery leaves (e.g. [1]), which are suggested to be adaptations to the high altitude tropical environment [7]. Once the plant has flowered and set seeds, it dies, leaving a tall hollow and dried-out stem. The seed capsules contain a huge number of tiny yellow seeds that can be easily dispersed by wind [8]. *L. rhynchopetalum* is most prominent and noticeable in the Afroalpine part of the Bale and Simien mountain systems, commonly within an altitudinal range of 3600–4500 m asl, where it serves as a tourist attraction. The significance of this species is, therefore, not only ecological but also recreational and economic. Despite its significance, it is one of the least studied lobelia species at a molecular level and, to our knowledge, no DNA sequence data from this species is available in GenBank. Little is known about its population genetics, which makes it difficult to conserve at its full range of genetic diversity in the presence of threats from fire and overgrazing. 

Fast evolving regions from nuclear and chloroplast genomes have been used to generate intraspecific DNA sequence data for plant population genetic studies [9–12], as it enables us to reveal the distribution of haplotypes, both within and among populations, and to identify species genetic diversity hotspots. 

In the present study, DNA sequence data from the internal transcribed spacers (*ITS*) of nuclear ribosomal DNA (rDNA) and eight chloroplast DNA (cpDNA) regions were generated from *L. rhynchopetalum *with the objectives of (1) population genetic and phylogenetic analyses for its conservation and evolutionary significance, (2) evaluating the utility of these DNA regions for population genetic and phylogenetic analyses, particularly within the genus *Lobelia*, and (3) contributing DNA sequence data from this species to Genbank so that it can be used for broader phylogenetic and phylogeographic analyses in combination with DNA sequence data from other *Lobelia* species.

## 2. Materials and Methods

### 2.1. Plant Material

A total of eight populations of *L. rhynchopetalum* collected from the Bale (6°48′N–7°08′N and 39°45′E–39°57′E) and Simien (13°05′N–13°25′N and 37°50′E-38°30′E) mountain systems were used in this study (Table 1). Goba-1, -2, and -3 populations were from the Bale mountains whereas Debark-1-5 populations were from the Simien mountains. Each population was represented by five individual plants. *Lobelia erinus* L. was included as an outgroup species for the phylogenetic analysis of *L. rhynchopetalum* populations and for comparative assessment of the DNA regions used in this study (see Table 2).

### 2.2. DNA Extraction

DNA was extracted from silica-gel-dried young leaves using a modified CTAB procedure as described in [13] except that 100 mg of fine powder of leaf material was used instead of 300 mg. DNA quality and concentration were measured using a Nanodrop ND-1000 spectrophotometer (Saveen Werner, Sweden).

### 2.3. PCR and Sequencing

Target DNA regions (Table 2) were amplified using a GeneAMP PCR system 9700 thermocycler with the following temperature profiles: initial 3 min denaturing at 94°C and final 7 min extension at 72°C with the intervening 30 cycles of 1 min denaturing at 94°C, 1 min primer annealing at 48°C, and 2 min primer extension at 72°C. The *ITS *was amplified and sequenced using *ITS5F* and *ITS4R* primers [14, Table 2]. Eighteen new primers were designed to amplify and sequence the* trnT*-*trnL*, *trn*f*M*-*trnS*, *petN*-*trnC*, *trnG*-*trnR*, *psbT*-*psbB*, *clpP intron-2*, *3′trnK*-*matK*, and *psbD*-*trnT* regions of cpDNA (Table 2) using the primer3 primer designing program [15]. The primers were designed to the conserved regions based on the aligned DNA sequences of *Trachelium caeruleum* L. (Campanulaceae) and *Helianthus annuus* L. (Asteraceae) (accession numbers *EU090187 *and *DQ383815*, resp.). These primers were named (Table 2) based on their 5′ position (forward primers) and 3′ position (reverse primers) in the *T. caeruleum* complete chloroplast genome sequence. 

The *trn*T*-trn*L intergenic spacer was amplified using primers *37258F *and *37820R*. The reverse primer, *37820R*, was used to sequence this region. The *trn*f*M*-*trnS* region contains the *trn*f*M* gene, *trn*f*M*-*trnG* intergenic spacer,* trnG* gene,* trnG*-*psbZ* intergenic spacer,* psbZ* gene,* psbZ*-*trnS* integenic spacer, and *trnS* gene in that order. This region was amplified in two segments with a combination of four primers (Table 2). The first pair of primers (*48992F *and *49584R*) amplified part of the *trn*f*M* gene and the full length of the* trnG* gene and the* trn*f*M*-*trnG* and the *trnG*-*psbZ* intergenic spacers. This part of the* trn*f*M*-*trnS* region was sequenced using primer *49584R*. The second pair of primers (*49595F* and *50079R*) amplified part of the *psbZ* gene and the* psbZ*-*trnS* intergenic spacer that were sequenced using primer* 50079R*. Similarly, part of the *petN*-*trnC* intergenic spacer was amplified and sequenced using primers *58099F *and *58955R*. 

Primers *10002F* and *10226R* were used to amplify the *trnG*-*trnR* intergenic spacer (Table 2). The complete sequence of this spacer was obtained using the forward primer, *10002F*. The *psbT*-*psbB* intergenic spacer was amplified using primers *26837F* and *27102R* and sequenced using the reverse primer,* 27102R*. Primers *111104F* and *111454R* were used to amplify* intron-2* (the intron between *exon-2 *and *exon-3*) of the *clpP* gene, whose partial sequence was obtained by using primer *111454R*. The *3′trnK-matK* portion of the *trnK* intron was amplified using primers *1825F* and *2195R,* and the amplified fragment was sequenced using primer *1825F*. Similarly, the *psbD*-*trnT* intergenic spacer was amplified using primers *53562F* and *54107R*. The reverse primer, *54107R*, was used to obtain the partial sequence of this spacer (Table 2). The PCR products were purified by QIAquick PCR purification kit (Qiagen GmbH, Germany) using a microcentrifuge as recommended by the manufacturer. Eight microlitre of purified PCR product (50–100 ng) was mixed with 2 *μ*L of 5 *μ*M sequencing primer and sent to the sequencing facility at the University of Oslo (http://www.bio.uio.no/ABI-lab/), where DNA sequencing was carried out. The representative nucleotide sequences of *ITS* and the eight cpDNA regions of *L. rhynchopetalum* were submitted to nucleotide sequence database (NCBI GenBank), and their accession numbers are given in Table 2.

### 2.4. Sequence Alignment and Data Analyses

DNA sequences were edited using BIOEDIT version 7.0.5 [16], and the quality of the sequences was visually inspected using Sequence Scanner version 1.0 (Applied Biosystems). Sequences were aligned using Clustal X version 1.81 [17]. PAUP* 4.0 Beta 10 [18] was used to construct a bootstrap 50% majority rule consensus tree based on Kimura distance coefficient [19]. Trees were generated using heuristic search with the tree-bisection-reconnection (TBR) branch swapping algorithm, and clade support was estimated using 1000 bootstrap replicates (starting trees were obtained via neighbor-joining, and initial Maxtree was set to 1000). Various population genetic analyses including gene and nucleotide diversity, analysis of molecular variance (AMOVA), haplotype distribution and interhaplotypic distance were conducted using Arlequin version 2 [20]. The minimum spanning tree (MST) of haplotypes was also generated using Arlequin.

## 3. Results

### 3.1. Some Sequence Characteristics of *L. rhynchopetalum*

In this study, full sequence length was obtained for the *ITSs* (*ITS-1*, *5.8S* and* ITS-2*),* trnG_GCC_* gene, and* trn*f*M*-*trnG_GCC_*, *trnG_GCC_-psbZ,* and *trnG_UCC_*-*trnR* intergenic spacers. Partial sequences from the *trnT*-*trnL*,* psbT*-*psbB*, *psbD*-*trnT*,* psbZ*-*trnS*,* petN*-*trnC* intergenic spacers, *trn*f*M*, *psbZ, *and* matK* genes, and *clpP intron-2* and 3′*trnK*-*matK* introns were also obtained. A total of 20 variable sites were obtained within *L. rhynchopetalum*, of which 3 are indel positions and the remaining 17 are substitutions. Indels were only obtained in the *ITS* and *trn*T-*trn*L regions. The *trnT*-*trnL* intergenic spacer sequences of all individuals from the Bale mountains were shorter by one nucleotide as compared to those from the Simien mountains. This single nucleotide long indel has clearly differentiated the populations according to their mountain system of origin. 

The number of variable sites for each region of *L. rhynchopetalum* determined after coding indels according to the simple indel coding method of Simmons and Ochoterena [21] is given in the 5th column of Table 2. The highest number of variable sites within* L. rhynchopetalum* was obtained from *ITS*, as expected. Eleven variable sites were obtained in the entire *ITS* region, nine within *ITS-1* and two within *ITS-2*. The* psbT*-*psbB*, *psbD*-*trnT*,* trn*f*M*-*trnS*, and *clpP intron-2* sequences were the least variable regions, as only a single variable site per region was obtained within *L. rhynchopetalum*. In the case of the *trn*f*M*-*trnS* region, the variable site was located within the *psbZ*-*trnS* intergenic spacer. The average percent variable sites (*%VS*) of cpDNA regions and *ITS* within *L. rhynchopetalum* were 0.4% and 1.4%, respectively. These values were increased to 9% and 28%, in that order when the* L. rhynchopetalum* sequence was aligned with that of *Lobelia erinus*. 

To evaluate the usefulness of the DNA regions used in this study for phylogenetic analysis, the DNA sequences of* L. rhynchopetalum* and *L. erinus* were aligned. Based on the aligned sequences of these species, different parameters of sequence variation of each DNA region were generated (Table 2). The %VS (without including indels) ranged from 2.1 (*trnG*-*trnR*) to 27.5 (*ITS*), of which 100% and 25.8% were parsimony informative, respectively. This analysis revealed a large number of parsimony informative variable sites in all DNA regions investigated in this study, with the exception of the *trnG*-*trnR* intergenic spacer (Table 2), suggesting their usefulness for phylogenetic analysis of the genus *Lobelia* and its various subgenera and sections.

### 3.2. Intrapopulation Genetic Analysis of *L. rhynchopetalum*

Genetic analyses of *L. rhynchopetalum* at the intra-population level were based on 20 polymorphic loci, which include both substitutions and indels. The number of haplotypes per population ranged from 1 to 5. No DNA sequence variation was obtained in the Goba-2, Goba-3, and Debark-2 populations. Since each of these populations carried a single haplotype, the estimates for their intra-population gene diversity and other related parameters were zero (see Table 1). Of the eight populations investigated in this study, Debark-1 was the most diverse, as the haplotype from each individual in the population was different. The haplotypes from the eight populations were in total 18, of which eight haplotypes were found in more than one population. Overall, 13 unique haplotypes were identified from the 40 individuals (Table 1). Five and eight of these haplotypes were unique to the Bale and Simien mountains, respectively.

Genetic diversity was estimated for each population and mountain system as gene diversity (GD; [22]) and nucleotide diversity (ND; [22, 23]). Nucleotide substitutions (transitions and transversions) are the major source of gene diversity within populations. The highest gene diversity (GD = 1.00) was obtained in the Debark-1 population with five haplotypes and six polymorphic sites, followed by Goba-1 (GD = 0.90) with 4 haplotypes and six polymorphic sites (Table 1). Population Goba-1 stood first in terms of nucleotide diversity (ND = 0.071), which is the average gene diversity over all loci under consideration (Table 1). Among the Simien mountain populations, the highest ND (0.038) was recorded in Debark-1. The estimates of ND were higher in the Bale mountains than in the Simien mountains (Table 1) while the estimates for GD were the same. Overall, GD and ND in *L. rhynchopetalum* were estimated to be 0.85 and 0.281, respectively. 

The mean interhaplotypic distance (MIHD) was calculated based on the Kimura distance method [19]. The highest estimate (0.081) was recorded in the Goba-1 population, which is almost twofold higher than the highest MIHD among the haplotypes in the populations from the Simien mountains. The mean number of pairwise differences (*π*) within populations ranged from 0.00 to 2.99, with the highest obtained in the Goba-1 population. The estimates for MIHD and *π* were higher within the Bale mountains than within the Simien mountains (Table 1). Tajima's test of selective neutrality (*D*; [24]) was also applied to each population and mountain system. The estimates of this parameter (*D*) ranged from −1.33 to 1.22 (Table 1), which is not significantly different from zero. We obtained a similar insignificant deviation from zero when each DNA region was considered separately (data not shown). Theta (*θ*), a central parameter in population genetic models, summarizes the rate at which mutation and random genetic drift generate and maintain variation within a given DNA region. The estimates of three *θ* estimators (*θ*_*π*_, *θ*_*S*_ and *θ*_*K*_) are given in Table 1. In this analysis, the estimate of *θ*_*π*_ was almost the same as that of π except the slight differences in standard deviation. The highest estimates for the three *θ* estimators were obtained in the Goba-1 population. 

### 3.3. Interpopulation Genetic Analysis of *L. rhynchopetalum*

**Figure 1:**
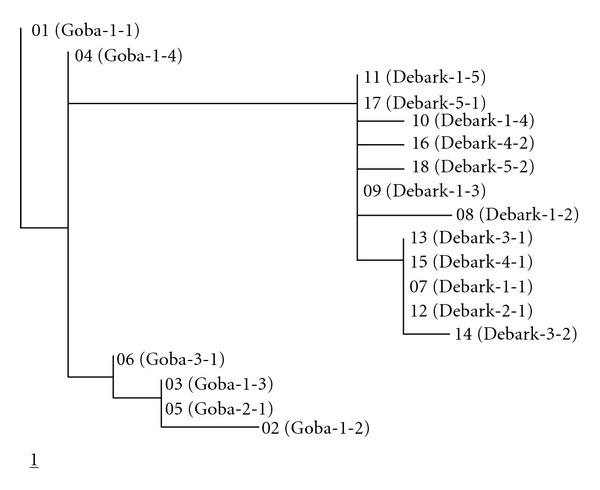
Minimum spanning tree (MST) of the 18 haplotypes generated from the ten *L. rhynchopetalum* populations. The MST was based on the K2p distance method. Numbers 01–18 are the haplotypes whereas the text in parenthesis is the population from which the haplotypes were generated. Note: haplotypes 03 and 05 are the same but in different populations; haplotypes 11 and 17 are the same but in different populations; haplotypes 07, 12, 13, and 15 are the same but in different populations.

**Figure 2:**
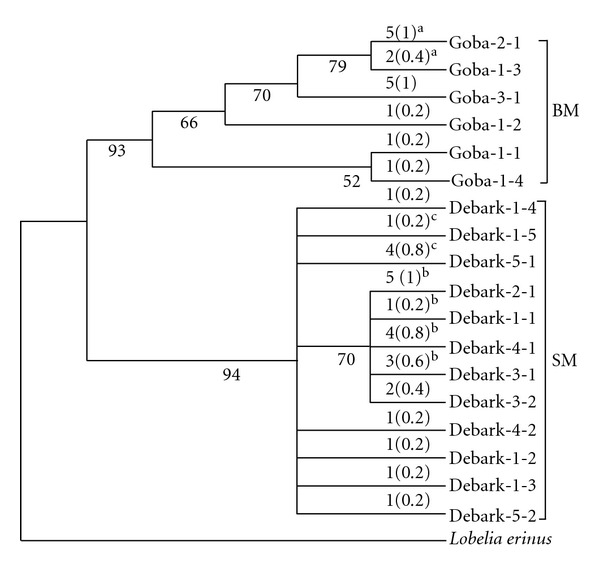
The bootstrap 50% majority rule consensus tree of 18 haplotypes from the ten *L. rhynchopetalum* populations. Bootstrap values greater than 50 are given below the branches. The frequency and relative frequency of each haplotype in each population are given above the branches outside and inside parenthesis, respectively. Haplotypes shared by more than one population are indicated with the same superscript.

We quantified the population differentiation of haplotypes by using the analysis of molecular variance model [25] for all populations and for populations from each mountain system. The differentiation between the Bale populations and between the Simien populations was similar (*F*_ST_ = 0.40). Overall, the analysis revealed that 85% of the variance in the distance matrix was accounted for by differences among populations and only 15% by diversity within populations (Table 3). When the significance of population differentiation was tested by 10000 permutations, populations were differentiated at a highly significant level (*P* < 0.001) within geographic locations and overall. The hierarchical AMOVA also revealed a significant geographic differentiation of *L. rhynchopetalum* populations. In addition, the minimum spanning tree (Figure 1) and the distance-based 50% bootstrap majority rule consensus tree (Figure 2) obtained from the analyses of the sequence data demonstrated a clear geographic differentiation of this species.

## 4. Discussion

### 4.1. The Utility of the DNA Regions for Population Genetic and Phylogenetics Studies

The presence of intraspecific variation in nuclear rDNA (e.g., [9, 11, 12]) and cpDNA (e.g., [10, 11, 26]) is well documented. Here, we used the internal transcribed spacers (*ITSs*) of nuclear rDNA and eight cpDNA regions for intra- and interpopulations genetic analyses of *L. rhynchopetalum*. In addition to information from published reports, our choice of the cpDNA regions used in this study was based on the aligned DNA sequences of *Trachelium caeruleum* and *Helianthus annuus*. The alignment of the sequences of these two species revealed mononucleotide repeat microsatellites and a large number of variable sites within the *trnT-trnL*,* trnG*-*trnR*, *psbT*-*psbB,* and *psbD*-*trnT* intergenic spacers, the 3′*trnK*-*matK* portion of the *trnK* intron, and the* clpP intron-2*. These regions were, therefore, targeted to identify polymorphic microsatellites within *L. rhynchopetalum*. However, no polymorphic microsatellites were found, and thus only variable sites due to nucleotide substitutions and indels were used. 

The *ITS* region has been commonly used for plant molecular systematics at lower taxonomic levels and for intraspecific genetic studies since it was first used in phylogenetic inference [27]. It has already become obvious that this region is much more variable than the fastest evolving regions of cpDNA. The result of this study is a further proof to this general understanding, as the *ITS* was over threefold more variable than the average variation obtained within the cpDNA regions in *L. rhynchopetalum*. The *psbD-trnT *region has been considered as one of the highly polymorphic regions of cpDNA [28] and proved to show some degree of polymorphism at the intraspecific level (e.g., [29]). The *trnT*-*trnL*, *3′trnK*-*matK*, *petN*-*trnC*, *psbT*-*psbB* (as part of *psbB*-*psbH*), and *trn*f*M*-*trnS* regions are also among the fast evolving cpDNA regions [28]. For example, the *trn*f*M*-*trnS* region was proved to be informative at the intraspecific level in *Eritrichium nanum* [30] and *Vigna angularis* [29]. The *trnG*-*trnR* intergenic spacer and the* clpP* intron were not part of the 34 fast evolving cpDNA regions reported in [28] and may not have been used for systematics and population genetic studies. 

The *trnG*-*trnR *region was revealed to be the least variable and parsimony informative in the aligned sequences of *L. rhynchopetalum* and *L. erinus* regardless of the fact that two variable sites were obtained within* L. rhynchopetalum* (see Table 2). On the other hand, when percent variable sites (*%VS*) and percent parsimony informative sites (*%PIS*) were considered, the *clpP intron 2* was found to be as informative as previously reported fast evolving cpDNA regions [28] and hence can be safely used for low taxonomic level phylogenetic studies. Generally, this analysis revealed that all DNA regions included in this study, with the exception of the *trnG*-*trnR* intergenic spacer, have a large number of parsimony informative variable sites, which can be used for phylogenetic analysis of the genus *Lobelia* and its various subgenera and sections, as exemplified using* L. erinus*. 

*L. rhynchopetalum* is more closely related to* Lobelia aberdarica* R. E. Fr. & T. C. E. Fr. than to most other east African giant lobelias, including *Lobelia gibberoa* Hemsl. [3]. Thirty polymorphic sites were obtained within a 650 bp *ITS* aligned sequence of *L. rhynchopetalum* and *L. aberdarica* (accession number, AF163435). The alignment of the 3′*trnK*-*matK* intron partial sequences (309 bp) of *L. rhynchopetalum* and *L. aberdarica* (accession number, AF176898) revealed ten potentially informative sites. Similarly a 489 bp *trnT*-*trnL* aligned partial sequence of *L. rhynchopetalum* and *L. giberroa* (accession number, DQ285239) revealed 18 potentially informative sites. Hence, the combination of these cpDNA regions could sufficiently resolve the phylogenetic relationships between the east African giant lobelias and beyond.

### 4.2. Intra- and Interpopulation Genetic Analysis of *L. rhynchopetalum*

To develop conservation strategies that preserve maximum levels of genetic diversity of *L. rhynchopetalum in situ* and make reasonable decisions about sampling procedures of germplasm for their *ex situ* conservation, one should know how its genetic variation is distributed within the species and what their population genetic structure looks like. The DNA sequence data from *ITS* and eight cpDNA regions proved to be a useful tool for this purpose and successfully applied to reveal the genetic diversity and population genetic structure of this endemic giant lobelia, regardless of limited number of variable sites obtained. Of the eight populations, two populations showed high genetic variation while three populations showed no variation within population. These populations were from both mountain systems, suggesting that the extent of within-population diversity is not limited to geographic regions. However, Simien mountains appeared to be a more favorable habitat for *L. rhynchopetalum* than the Bale mountains, as four of its five populations showed intrapopulation haplotypic diversity.

Many natural ecosystems are subject to habitat fragmentation, which results in smaller and more isolated populations. Plant species remaining in fragmented habitats are of conservation concern due to impacts of decreased population size and increased isolation that threaten their viability due to genetic drift and consequently lower genetic diversity (e.g. [31]). The consequences of such isolation likely increase inbreeding, and in turn greater exposure to genetic drift, resulting in loss of genetic diversity. The absence of genetic variation in some *L. rhynchopetalum *populations may be best explained by genetic drift in relation to their small population size and limited gene flow between populations, as small isolated populations are highly likely to diverge from each other due to genetic drift, which causes fixation of alleles. 

Population differentiation is driven by various evolutionary forces such as mutation, gene flow, genetic drift, and selection, and its extent depends on the relative strength of these individual forces in interaction with life history traits of the species. Theta (*θ*) is a central parameter in population genetic models for the balance between mutation and random genetic drift. For haplotypic data, theta is measured as follow *θ* = 2*N*_*e*_ *μ*, where *N*_*e*_ is effective population size and *μ* is a mutation rate per nucleotide site under neutral evolution theory model [32]. Thus, *θ* summarizes the rate at which mutations and random genetic drift generate and maintain variation within a given DNA region, under conditions in which natural selection is not operating. There were only two variants per polymorphic site in our aligned DNA sequence data set, which fits to the assumption of the infinite-site model. Pi (π) is the other measure of sequence variability, which measures the pairwise differences between sequences. In this study, the estimates for *θ*_*π*_ and *π* are similar. This suggests that the data fits with the infinite-site model, as these two parameters have equal expectation under this model (see [32]). Tajima's *D* test is the test for selective neutrality, and the parameter (*D*) is considered to be zero for neutral loci [24]. In our analysis, *D* was not significantly different from zero in all populations and groups. Therefore, we conclude that the neutral mutation hypothesis explains the DNA sequence variation obtained in this study. 

Thirteen unique haplotypes were obtained by analyzing 40 individual plants. Only three of these, haplotypes were shared among populations of the same mountain systems. Analysis of molecular variance revealed that a high proportion (85%) of the genetic variation was found among populations, and within-population variation only accounted for 15% of the total variation. The 9 variable sites obtained from cpDNA regions were the major source of the highly significant population differentiation at all hierarchical levels, as only three of these sites were variable at the intrapopulation level. Such high population differentiation is not uncommon in endemic species with limited distribution and small population sizes, as a consequence of the pronounced effects of genetic drift (e.g., [33]). When populations are differentiated at a highly significant level, it is recommended to conserve representative samples *ex situ* from each population to reduce the risk of losing unique genetic variants. For *in situ *conservation, priority should be given to populations with relatively high genetic diversity. The results of this study and the ISSR-based study [34] are not in complete agreement as to which of these populations have high diversity, which makes it difficult to prioritize specific populations for *in situ* conservation. However, simultaneous consideration of the two data sets and environmental factors suggests Goba-1 and Debark-1 as good candidates for *in situ* conservation. 

AMOVA revealed a highly significant differentiation not only between populations but also among the two mountain systems (Table 3). The differentiation of populations according to geographic areas was also clearly revealed in the 50% bootstrap majority rule consensus tree (Figure 2). Two major clades supported by high bootstrap values (>90%) were formed, in which the haplotypes from the two mountain systems were clearly separated. The result is in agreement with the ISSR-based study [34] in grouping populations according to mountain system of origin. Generally, all analyses revealed a significant differentiation of *L. rhynchopetalum* populations at various hierarchical levels. Such a high population differentiation can be partly explained by limited gene flow. Several factors, such as geographic distance between populations, pollen and seed dispersal mechanisms, and mode of reproduction, have a direct impact on the extent of gene flow between populations. For example, the significant differentiation between the two mountain systems can be partly explained by the Rift Valley as a barrier to gene flow. Similar results were previously reported in *L. giberroa* between these mountain systems [35]. Such a limited gene flow between populations allows further population differentiation, which could lead to speciation. 

Knox and Palmer [3] suggested that giant lobelias appear to have initially colonized the ancient upland in East Africa and then moved onto the tall mountains as they arose. *Lobelia acrochila* (E. Wimm.) Knox is the most closely related species to *L. rhynchopetalum* [3, 36]. The inclusion of *L. acrochila* in such type of studies will shed more light to the evolutionary radiation of giant lobelias in eastern Africa. The fact that the Simien mountains are the northernmost end of the distribution of giant lobelias in eastern Africa and the suggestion that giant lobelias were expanding from south towards north [35] may give more weight to the Bale mountains, than to the Simien mountains, as a likely place for the origin of this species. However, since populations from other mountains where this species may be found (though to a lesser extent) were not included in this study, further studies by including these populations are needed to support this suggestion. 

This work has revealed the existing haplotypic variation and population genetic structure of *L. rhynchopetalum*. The combination of deterministic and stochastic factors and factors affecting gene flow seems to have played a significant role for the highly significant differentiations of the species at different hierarchical levels. With about 85% of the total genetic variation residing in between populations, each population should be regarded as an important contributor to the overall amount of genetic variation and a significant unit for conservation efforts of this species. Our recommendation is that representative populations from different altitudes and geographic locations should be targeted for conservation purposes, as it reduces the risk of losing unique genetic variants due to several factors. The DNA sequence data generated and submitted to GenBank and the nine pairs of new primers that can be applied to any *Lobelia* and other closely related species, both for population genetic and phylogenetic studies, are also a significant contribution of this work. Furthermore, since the cpDNA primers were designed based on the aligned sequences of *Trachelium caeruleum* (Campanulaceae) and *Helianthus annuus* (Asteraceae), they may be useful for similar studies not only in lobelias but also in various Campanulaceae and Asteraceae species.

## Figures and Tables

**Table 1:** Genetic analysis of *L. rhynchopetalum* populations from the Bale mountains (BM) and Simien mountains (SM) at different hierarchical levels.

Population/group	Location-mountains	Altitude (m asl)	NGC /SS	NH	NPS	GD	ND	MIHD ± SD	*π*	*θ*_*π*_	*θ*_*S*_	*θ*_K_*	*D*
Goba-1^ad^	15 km from Goba to Mena-BM	4000–4300	5	4	5	0.90 ± 0.16	0.071 ± −0.052	0.081 ± 0.045	2.99 ± 1.87	2.99 ± 2.19	2.40 ± 1.51	7.11 [1.54, 33.08]	1.12
Goba-2^a^	16.8 km from Goba to Mena-BM	4000–4300	5	1	0	0.00 ± 0.00	0.000 ± −0.000	0.000 ± 0.000	0.00 ± 0.00	0.00 ± 0.00	0.00 ± 0.00	0.00 [0.00, 0.00]	0.00
Goba-3	18.5 km from Goba to Mena-BM	4000–4300	5	1	0	0.00 ± 0.00	0.000 ± 0.000	0.000 ± 0.000	0.00 ± 0.00	0.00 ± 0.00	0.00 ± 0.00	0.00 [0.00, 0.00]	0.00
Debark-1^bcdef^	47 km from Debark to Sankaber-SM	3600–3800	5	5	6	1.00 ± 0.12	0.038 ± 0.031	0.042 ± 0.031	1.68 ± 1.17	1.68 ± 1.37	1.92 ± 1.27	—	−1.09
Debark-2^b^	48 km from Debark to Sankaber-SM	3600–3800	5	1	0	0.00 ± 0.00	0.000 ± 0.000	0.000 ± 0.000	0.00 ± 0.00	0.00 ± 0.00	0.00 ± 0.00	0.00 [0.00, 0.00]	0.00
Debark-3^be^	51 km from Debark to Sankaber-SM	3600–3800	5	2	1	0.60 ± 0.17	0.014 ± 0.015	0.024 ± 0.024	0.61 ± 0.57	0.61 ± 0.67	0.48 ± 0.48	0.69 [0.15, 3.19]	1.22
Debark-4^b^	53 km from Debark to Sankaber-SM	3600–3800	5	2	2	0.40 ± 0.24	0.019 ± 0.019	0.048 ± 0.034	0.83 ± 0.70	0.83 ± 0.81	0.96 ± 0.76	0.69 [0.15, 3.18]	−0.97
Debark-5^cf^	54.5 km from Debark to Sankaber-SM	3600–3800	5	2	1	0.40 ± 0.24	0.009 ± 0.012	0.024 ± 0.024	0.41 ± 0.44	0.41 ± 0.52	0.48 ± 0.48	0.69 [0.15, 3.18]	−0.82
Bale			15	5	5	0.70 ± 0.08	0.035 ± 0.025	0.071 ± 0.042	1.48 ± 0.95	1.48 ± 1.06	1.54 ± 0.85	2.20 [0.77, 5.97]	−0.28
Simien			25	8	9	0.70 ± 0.08	0.025 ± 0.018	0.046 ± 0.032	1.11 ± 0.75	1.11 ± 0.83	1.85 ± 0.89	3.67 [1.57, 8.21]	−1.33
All			40	13	20	0.85 ± 0.04	0.28 ± 0.15	0.201 ± 0.062	8.23 ± 4.29	8.23 ± 4.32	3.99 ± 2.16	6.29 [3.19, 12.06]	0.81

NGC/SS: number of gene copies/sample size; NH: number of haplotypes; NPS: number of polymorphic sites; GD: gene diversity; ND: nucleotide diversity (based on polymorphic loci only); MIHD: mean interhaplotypic distance (based on Kimura 2P method); SD: standard deviation (for both the sampling and the stochastic processes); *π*: mean number of pairwise difference (based on polymorphic loci only). Populations sharing the superscript a, b, or c have some haplotypes in common. Populations sharing the superscript d, e, or f were not significantly different from each other when tested using global test of population differentiation, based on Markov chain length of 10000 (significance level = 0.05). *D*: Tajima's test of selective neutrality. *θ*_*π*_, *θ*_*S*_, and *θ*_*K*_ are different estimators of theta (*θ*). *: Values in the square brackets are 95% confidence interval limits around *θ*_*K*_. Note: for analysis at mountain-system level, all individuals of the same category were pooled together as a single unit.

**Table 2:** (1) Name and sequence of primers used for the amplification and sequencing of target DNA regions; (2) Genbank accession numbers of representative sequences and some sequence characteristics of each DNA region.

Target region	Primer name	Primer sequence	GBAN	NVSWLr	TAL^e^	NVS^e^	%VS^e^	NPIS^e^	%PIS^e^	%PIVS^e^
*ITS* ^ a ^	*ITS5F* ^ c^ *ITS4R* ^ d^	5′-GGAAGGAGAAGTCGTAACAAGG-3′ 5′-TCCTCCGCTTATTGATATGC-3′	FJ664108-9	11	719	198	27.5	51	7.1	25.8

*trn*T_UGU_ *-trn *L_UAA_^b^	37258F^d^ 37820R^c^	5′-TGCAATGCTCTAACCTCTGA-3′ 5′-CGATTTTATCATTTATCTATCTCCAA-3′	FJ664110-1	2	525	47	9.0	15	2.9	31.9

*trn*fM_CAU_-*trn *S_UGA_^b^	48992F^d^ 49584R^c^ 49595F^d^ 50079R^c^	5′-GTAGCTCGCAAGGCTCATAAC-3′ 5′-TTCTGGTGGGTATCCTTAATTCTC-3′ 5′-ATACCCACCAGAAAGACTAATCCA-3′ 5′-CCATCTCTCCGAAAGACAATTTTA-3′	FJ664119-20	1	976	134	13.7	31	3.2	23.1

*pet*N-*trn *C_GCA_^b^	58099F^c^ 58955R^c^	5′-CCCAAGCGAGACTTACTATATCCA-3′ 5′-AAATCCTTTTTCCCCAGTTCAA-3′	FJ664115-6	3	291	20	6.9	12	4.1	60.0

*trn*G_UCC_-*trn *R_UCU_^b^	10002F^c^ 10226R^d^	5′-CTAGCCTTCCAAGCTAACGATG-3′ 5′-GACCTCTGTCCTATCCATTAGACAAT-3′	FJ664121-2	2	195	4	2.1	4	2.1	100.0

*psb*T-*psb*B^b ^	26837F^d^ 27102R^c^	5′-GAATGTATAAACCAATGCTTCC-3′ 5′-GAATTTGGAGCATTCCAAAAACT-3′	FJ664118	1	205	18	8.8	8	3.9	44.4

*Clp*P *intron 2 * ^b ^	111104F^d^ 111454R^c^	5′-GCCTTCGCCATATGAAA-3′ 5′-ATGATGGCTCCGTTGCT-3′	FJ664112	1	311	36	11.6	11	3.5	30.6

*3 *′*trn*K_UUU_ *-mat*K^b^	1825F^c^ 2195R^d^	5′-CGGAACTAGTCGGATGGAGT-3′ 5′-GCTTCTTCTATTTCGCGTAGGT-3′	FJ664113	2	307	26	8.5	16	5.2	61.5

*psb*D-*trn *T_GGU_^b^	53562F^d^ 54107R^c^	5′-TGATCTGTAATCAAAGCAAGATAGTGA-3′ 5′-CGGTAGAGTAAGCCCATGGTA-3′	FJ664117	1	468	43	9.2	16	3.4	37.2

^a^Primers' original reference is White et al. [14]; ^b^primers were designed for this study; ^c^primers were used both for amplification and sequencing; ^d^primers were used for amplification only; ^e^the values were calculated based on aligned sequences of *Lobelia rhynchopetalum* and *Lobelia erinus*. GBAN: gene bank accession numbers of *L. rhynchopetalum* sequences. Note: in cases when there is more than one accession number for a given DNA region, the accession numbers are given in range. For example, FJ664108-9 represents two accession numbers (FJ664108 and FJ664109). NVSWLr: number of variable sites within *L. rhynchopetalum*; TAL: total aligned length; NVS: number of variable sites; %VS: percent variable sites; NPIS: number of parsimony informative sites; %PIS: percent parsimony informative sites; %PIVS: percent parsimony informative variable sites.

**Table 3:** Analysis of molecular variance (AMOVA) at different levels based on the Kimura K2P distance method.

Group	Source of variations	df	Sum of squares	Variance components	% variations	Fixation index
The Bale populations	AP	2	4.38	0.34Va	40.42	*F*_ST_: 0.40*
	WP	12	5.98	0.49Vb	59.58	
	Total	14	10.36	0.84		

The Simien populations	AP	4	6.16	0.24Va	39.89	*F*_ST_: 0.40*
	WP	20	7.13	0.36Vb	60.11	
	Total	24	13.29	0.59		

All populations	AP	7	95.20	2.63Va	85.39	*F*_ST_: 0.85*
	WP	32	14.40	0.45Vb	14.61	
	Total	39	109.60	3.08		

Two mountains^a^	AM	1	84.3	4.46Va	87.01	*F*_ST_: 0.87*
	WM	38	25.3	0.66Vb	12.99	
	Total	39	109.6	5.12		

Two geographic groups	AG	1	84.29	4.39Va	85.87	*F*SC: 0.37*
	APWG	6	10.91	0.27Vb	5.34	*F*ST: 0.91*
	WP	32	14.40	0.45Vc	8.79	*F*CT: 0.86*
	Total	39	109.60	5.12		

*P-value < 0.001 (significance test at 10000 permutations). AP: among populations; WP: within populations; AM: among mountains; WM: within mountains; AG: among groups; APWG: among populations within groups. ^a^Analysis was based on sequences pooled from individuals within the same mountain system.
